# CD133^+^CD24^lo^ defines a 5-Fluorouracil-resistant colon cancer stem cell-like phenotype

**DOI:** 10.18632/oncotarget.12168

**Published:** 2016-09-21

**Authors:** Amy V. Paschall, Dafeng Yang, Chunwan Lu, Priscilla S. Redd, Jeong-Hyeon Choi, Christopher M. Heaton, Jeffrey R. Lee, Asha Nayak-Kapoor, Kebin Liu

**Affiliations:** ^1^ Department of Biochemistry and Molecular Biology, Medical College of Georgia, Augusta University, Augusta, GA 30912, USA; ^2^ Georgia Cancer Center, Augusta University, Augusta, GA 30912, USA; ^3^ Charlie Norwood VA Medical Center, Augusta, GA 30904, USA

**Keywords:** CD133, CD24, colon cancer stem cells, 5-Fluorouracil

## Abstract

The chemotherapeutic agent 5-Fluorouracil (5-FU) is the most commonly used drug for patients with advanced colon cancer. However, development of resistance to 5-FU is inevitable in almost all patients. The mechanism by which colon cancer develops 5-FU resistance is still unclear. One recently proposed theory is that cancer stem-like cells underlie colon cancer 5-FU resistance, but the phenotypes of 5-FU-resistant colon cancer stem cells are still controversial. We report here that 5-FU treatment selectively enriches a subset of CD133^+^ colon cancer cells *in vitro*. 5-FU chemotherapy also increases CD133^+^ tumor cells in human colon cancer patients. However, sorted CD133^+^ colon cancer cells exhibit no increased resistance to 5-FU, and CD133 levels exhibit no correlation with colon cancer patient survival or cancer recurrence. Genome-wide analysis of gene expression between sorted CD133^+^ colon cancer cells and 5-FU-selected colon cancer cells identifies 207 differentially expressed genes. CD24 is one of the genes whose expression level is lower in the CD133^+^ and 5-FU-resistant colon cancer cells as compared to CD133^+^ and 5-FU-sensitive colon cancer cells. Consequently, CD133^+^CD24^lo^ cells exhibit decreased sensitivity to 5-FU. Therefore, we determine that CD133^+^CD24^lo^ phenotype defines 5-FU-resistant human colon cancer stem cell-like cells.

## INTRODUCTION

The chemotherapeutic agent 5-Fluorouracil (5-FU), in combination with other cytotoxic or targeted agents, is a standard adjuvant therapy for certain human patients with stage 2 colorectal cancer and almost all patients with stages 3 and 4 colorectal cancer [1–3]. However, development of cancer cell resistance to 5-FU is almost inevitable in colorectal cancer patients [4], resulting in tumor recurrence and metastasis to distant organs, primarily to the liver, which accounts for over 90% of human colorectal cancer mortality [5]. The mechanism underlying colorectal cancer 5-FU resistance is largely unknown. Recent studies suggests that the 5-FU-resistant colorectal cancer cells represent a subset of tumor cells in the original tumor population that are phenotypically and functionally distinct from the majority of the tumor cell population. These cells are referred to as colon cancer stem cells (CSC) or colon cancer-initiating cells (C-IC) [6–10]. 5-FU is an S phase-specific cytotoxic agent that selectively targets rapidly proliferating cells, such as tumor cells. CSCs or C-ICs, including colorectal CSCs, are quiescent or slowly cycling cells; 5-FU may therefore kill the rapidly dividing cancer cell population to enrich the rare subsets of colorectal CSCs. Indeed, recent studies have shown that 5-FU treatment selectively enriches subsets of colon cancer cells with colon CSC phenotypes [11–16].

Emerging experimental data suggest the majority of colon tumors contain subpopulations of CSCs or C-ICs [17–19]. CD133 is the most commonly used marker for human colorectal CSCs [8, 9, 20–28]. CD44 is a major hyaluronan receptor that has been extensively studied as another human colorectal CSC marker [18, 23, 27–34]. In addition, several other markers, including CD24 [27, 35], CD26 [17] and CD166 [19, 27, 29, 31, 36] have been identified as potential human colon CSC markers. However, although these colorectal CSC markers have been identified and characterized extensively in both cell lines and tumor tissues, no consensus has been reached regarding the general phenotypes that define human colorectal CSCs [29], as these markers are reversible and may vary according to tumor types, stages and chemotherapies [14, 26, 37]. Furthermore, the phenotypes of CSCs, including colorectal CSCs, have significant plasticity that is driven by not only intrinsic factors but also by the dynamic changes in the tumor microenvironment [6, 38–41]. New studies are continuously reported to further define colorectal CSC phenotypes, mainly through combinations of markers [15, 27, 28, 42–45].

Although colorectal CSC markers have been studied as biomarkers for colorectal cancer prognosis [20, 21, 24, 25, 46, 47], the most clinically relevant value of CSCs, including colorectal CSCs, is perhaps their putative role as the origin of chemoresistance [11–15, 48]. The first indication that colorectal CSCs are responsible for resistance to 5-FU came from studies of isolated primary colon cancer cells. Isolated CD133^+^ primary human colon cancer cells were observed to be highly resistant to 5-FU, whereas the unpurified cancer cells or purified CD133^−^ cells from the same human colon cancer specimens showed a high sensitivity to 5-FU [49]. However, it seems that not all CD133^+^ colon cancer cells are colon CSCs since only approximately 1 out of 262 CD133^+^ colon tumor cells are estimated to be colon CSCs [8]. We therefore hypothesize that a subset of CD133^+^ human colon cancer cells are 5-FU-resistant colon CSCs and carried out this study to test this hypothesis. To this end, we determined that a subset of CD133^+^CD24^lo^ colon cancer cells represent colon CSCs that underlie colon cancer 5-FU resistance.

## RESULTS

### 5-FU selectively enriches CD133^+^ colon cancer cells *in vitro*

**Figure 1:**
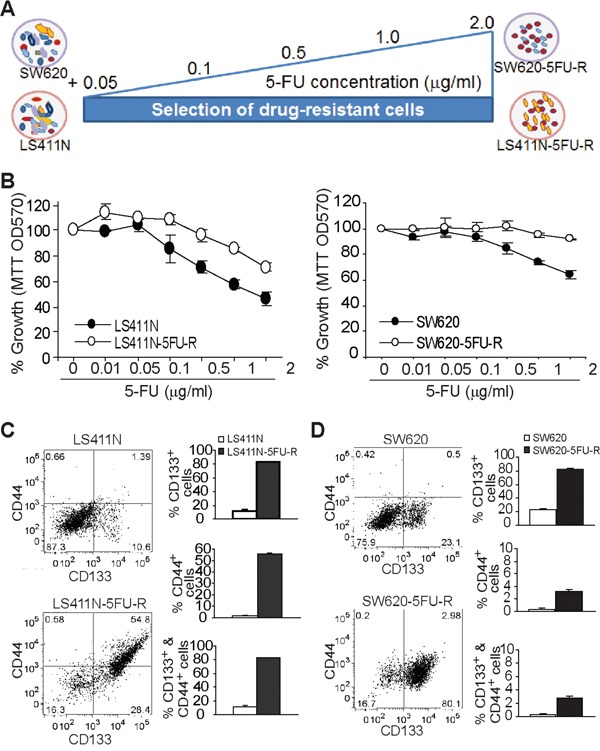
Enrichment of CD133^+^ cancer stem-like cells is linked to 5-FU resistance of human colon cancer **A.** Scheme of establishment of 5-FU-resistant colon cancer cell lines. Human colon carcinoma cell lines SW620 and LS411N were cultured in the presence of increasing 5-FU concentrations as indicated to establish 5-FU-resistant SW620-5FU-R and LS411N-5FU-R cell lines, respectively. **B.** The parent (LS411N and SW620) and the respective 5-FU-resistant human colon carcinoma cells (LS411N-5FU-R and SW620-5FU-R) were cultured in the presence of 5-FU at the indicated doses for 3 days and analyzed for growth using MTT assays. **C** and **D**. The parent and 5-FU-resistant cell lines were analyzed for cell surface CD133 and CD44 protein levels by flow cytometry. Left panels are plots of CD133 and CD44 levels, and the right panels are quantification of cell surface CD133 and CD44 protein levels of the indicated cell lines.

Human colon carcinoma LS411N and SW620 cells were cultured in the presence of increasing concentrations of 5-FU. Cells that survived 2.0 μg/ml 5-FU were established as cell lines and termed LS411N-5FU-R and SW620-5FU-R, respectively (Figure 1A). The 5-FU-resistant cell lines were then compared to the respective parent cell lines for their sensitivity to 5-FU. Both LS411N-5FU-R and SW620-5FU-R cells exhibited decreased sensitivity to 5-FU as compared to the respective parent cell lines (Figure 1B). Analysis of cell surface markers revealed that the majority of LS411N-5FU-R cells are CD133^+^CD44^+^ (Figure 1C), whereas the majority of SW620-5FU-R cells are CD133^+^CD44^−^. These observations suggest that CD133^+^ and/or CD44^+^ colon cancer cells are associated with 5-FU resistance.

### CD133 and CD44 expression are not correlated with sphere formation potential in colon cancer cells

CD133 and CD44 have been proposed as markers of colon CSCs [10, 18]. We sought to determine the relationship between CD133/CD44 expression and colon cancer cell sphere formation potential. Seven human colon cancer cell lines were analyzed for CD133 and CD44 expression on their surface. CD133^+^ and CD133^+^CD44^+^ cells are rare populations in all seven cell lines. In contrast, more than 37% of cells are CD44^+^ in four of the seven human colon carcinoma cell lines (Supplementary Figure S1A and S1B). Analysis of sphere formation potential of the seven cell lines revealed that three cell lines (LS411N, SW620 and T84) possess high sphere formation capability, whereas the other four cell lines exhibit very low sphere formation capability (Supplementary Figure S1C).

### 5-FU therapy increases CD133^+^ tumor population in human colon cancer patients

**Figure 2:**
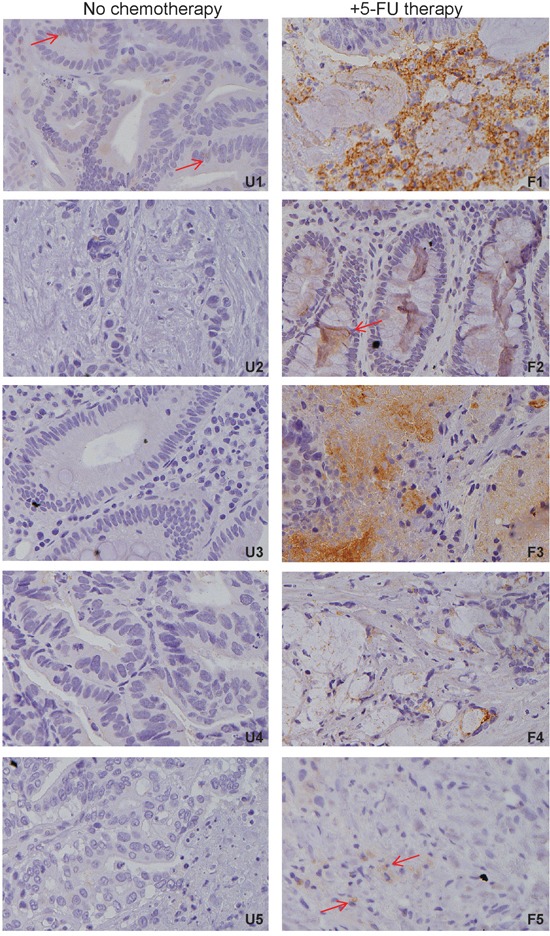
5-FU chemotherapy enriches CD133^+^ cancer cells in human colon cancer patients Tumor tissues from human colon cancer patients without prior 5-FU therapy (n=5) and with prior 5-FU therapy (n=5) were stained with CD133-specific antibody. Brown color indicates CD133 protein expression, with counterstaining by hematoxylin in blue. Shown are representative images of colon carcinoma tissues from each of the five patients without prior 5-FU therapy (U1-5) and with 5-FU therapy (F1-5). Shown is CD133 staining intensity. Red arrows indicate CD133^+^ cells.

To determine whether the observation that 5-FU treatment selectively enriches CD133^+^ colon cancer cells *in vitro* can be extended to human colon cancer patients, colon carcinoma tissues from human patients with and without prior 5-FU therapies were stained with CD133-specific antibodies and analyzed for CD133 protein expression. One of the five colon carcinoma tissues from human patients without 5-FU therapy expresses CD133, albeit at a low level. The remaining four tumor tissues have no detectable CD133 (Figure 2 U1-5). In contrast, four of the five colon carcinoma tissues from human patients treated with 5-FU have high levels of CD133 protein (Figure 2 F1-5). Therefore, 5-FU therapy enriches CD133^+^ colon cancer cells in human colon carcinoma tissues.

### CD133 protein level is not a prognostic marker in human colorectal cancer

The above observations indicate that 5-FU enriches CD133^+^ tumor cells in human colon cancer patients. Because almost all human colon cancer patients inevitably develop resistance to 5-FU therapy, we next sought to determine whether CD133^+^ tumor cells are correlated with human colorectal cancer patient disease outcomes. CD133 protein levels were analyzed in tumor specimens from 147 colorectal cancer patients and correlated to disease outcomes. No correlation was observed between CD133 protein levels and patient survival (Supplementary Figure S2A) or cancer recurrence (Supplementary Figure S2B).

### CD133 alone does not define a 5-FU-resistant phenotype

**Figure 3:**
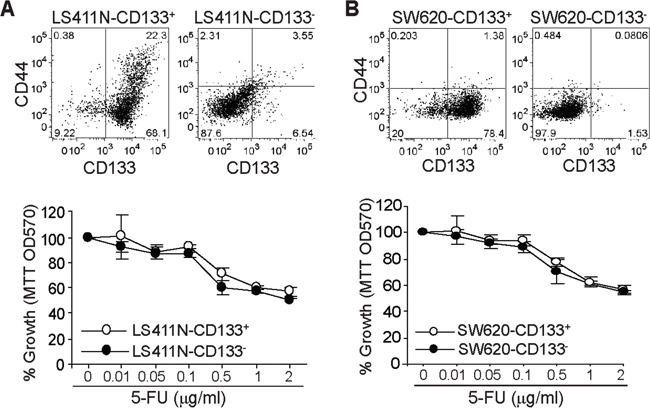
CD133^+^ colon cancer cells and 5-FU sensitivity CD133^+^ and CD133^−^ cells were sorted from LS411N **A.** and SW620 **B.** cells, respectively. The cells were stained with CD133- and CD44-specific antibodies and analyzed by flow cytometry (top panel). The two subsets of cells were cultured in the presence of 5-FU at the indicated concentrations for 3 days and analyzed for viability by MTT assays.

The above observations indicate that 5-FU enriches CD133^+^ human colon cancer cells both *in vitro* and *in vivo*, but CD133 alone does not define the colorectal cancer disease outcome. To determine whether CD133^+^ cells are responsible for 5-FU resistance, CD133^+^ and CD133^−^ cells were sorted from the parent LS411N (Figure 3A) and SW620 (Figure 3B) cells, respectively to establish four cell sublines: LS411N-CD133^+^, LS411N-CD133^−^, SW620-CD133^+^ and SW620-CD133^−^. The four sublines of cells were then tested for their sensitivity to 5-FU. Analysis of cell viability indicates that CD133^+^ colon cancer cells and CD133^−^ colon cancer cells derived from both human colon cancer cell lines exhibit no differences in their sensitivity to 5-FU (Figure 3A and 3B).

### Genome-wide gene expression profiles of 5-FU-resistant human colon cancer cells

**Figure 4:**
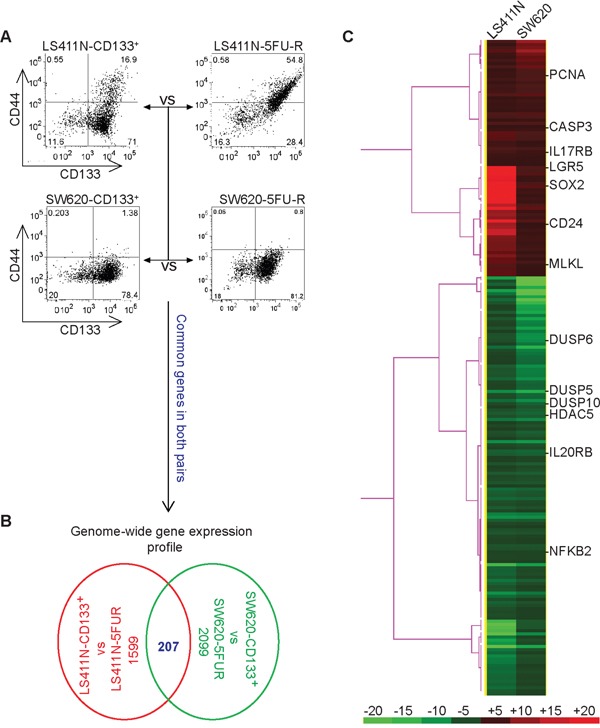
Genome-wide gene expression profiles of colon cancer stem-like cells **A.** Scheme of genome-wide gene expression analysis. LS411N-CD133^+^ cells were compared to LS411N-5FU-R cells and SW620-CD133^+^ cells were compared to SW620-5FU-R cells by DNA microarray analysis. Shown are phenotypes of the two pairs of cells. **B.** Analysis of the differentially expressed genes between CD133^+^ cells and 5-FU-R cells. Genes whose expression levels are changed by at least 2 fold (either up-regulated or down-regulated) were selected. The ratios are LS411N-CD133^+^/LS411N-5FUR and SW620-CD133^+^/SW620-5FU-R. The number of differentially expressed genes of each pair and the commonly differentially expressed genes of the two pairs of cells are shown. **C.** The commonly differentially expressed genes in two pairs of cells as shown in A and B (n=207) was selected. Cluster 3.0 program was used to analyze the gene expression patterns in a one-dimensional hierarchical clustering to generate gene dendrograms based on the pair-wise calculation of the Pearson coefficient of normalized fluorescence ratios as measurements of similarity and linkage clustering. The clustered data were loaded into TreeView program and displayed by the graded color scheme. Genes that have known functions in stem cell maintenance (CD24, LGR5, and SOX2), cell proliferation, and death (PCNA, CASP3, MLKL, DUSP5, DUSP6, NFKB2, and HDAC5), and for immune response (IL17RB and IL20RB) are indicated. Red columns indicate genes whose expression level is higher in LS411N-CD133^+^ and SW620-CD133^+^ cells as compared to LS411N-5FU-R and SW620-5FU-R cells, respectively. Green columns indicate genes whose expression level is higher in LS411N-5FU-R and SW620-5FU-R cells as compared to LS411N-CD133^+^ and SW620-CD133^+^ cells, respectively. The color bar at the bottom panel represents the level of differential expression. The number above the bar indicates the fold changes.

Both LS411N-5FU-R and SW620-5FU-R cells are CD133^+^ and resistant to 5-FU (Figure 1). However, while both LS411N-CD133^+^ and SW620-CD133^+^ cell lines are CD133^+^, they are still as sensitive to 5-FU as CD133^−^ cells (Figure 3). Therefore, we reasoned that LS411N-5FU-R and SW620-5FU-R represent only small subsets of CD133^+^ cells in LS411N-CD133^+^ and SW620-CD133^+^ cell lines. To identify these subsets of 5-FU-resistant CD133^+^ cells, we set up two comparisons: LS411N-CD133^+^ vs LS411N-5FU-R and SW620-CD133^+^ vs SW620-5FU-R. We then performed genome-wide gene expression analysis (Figure 4A). The rationale is that differentially expressed genes may define the 5-FU-resistant subsets of cells. A total of 1599 and 2099 genes were differentially expressed between LS411N-CD133^+^ and LS411N-5FU-R and between SW620-CD133^+^ and SW620-5FU-R cells (respectively) by a 2 fold cutoff. Among these genes, 207 genes are differentially expressed in both pairs of cells (Figure 4B). Some of these 207 genes (such as LGR5 and SOX2) have known functions in stem cell maintenance. Other genes have known functions in cellular proliferation and death (including PCNA, DUSO5, DUSP6 and DUSP10, NFKB2, CASP3 and MLKL), whereas two genes (IL17RB and IL20RB) function in immune response and inflammation (Figure 4C). Interestingly, the expression level of CD24, a gene whose absence/lower expression level is used as a CSC marker in breast cancer but whose high expression level is proposed as a CSC marker in colon cancer cells [23, 33, 35, 36, 50–53], is lower in both LS411N-5FU-R and SW620-5FU-R cells than that in LS411N-CD133^+^ and SW620-CD133^+^ cells (Figure 4C).

### CD133^+^CD24^lo^ colon cancer cells exhibit decreased sensitivity to 5-FU

**Figure 5:**
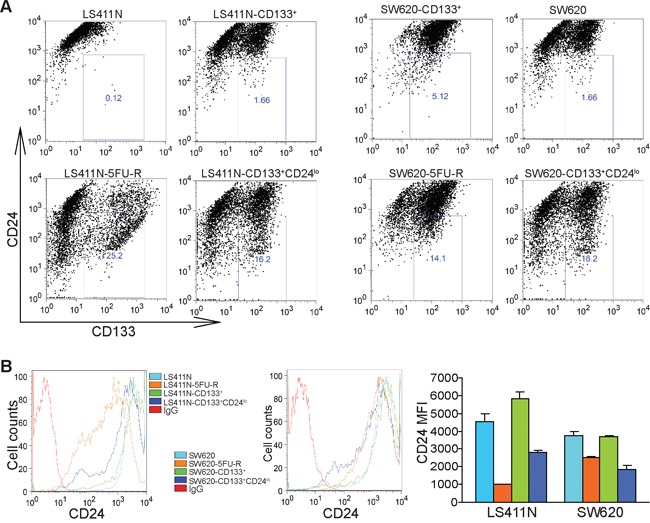
CD133^+^CD24^lo^ cells phenotypically resemble 5-FU-resistant human colon carcinoma cells **A.** CD133 and CD24 expression profiles in subsets of colon cancer cells. The indicated four subsets of LS411N and SW620 cells were stained with CD133- and CD24-specific antibodies and analyzed by flow cytometry. Shown are representative images of one of two experiments. **B.** CD24 protein levels as shown in A were plotted as to show mean fluorescent intensity (MFI). The MFI of the four subsets of LS411N and SW620 cells were quantified and presented at the right. Column: mean; Bar:SD.

**Figure 6:**
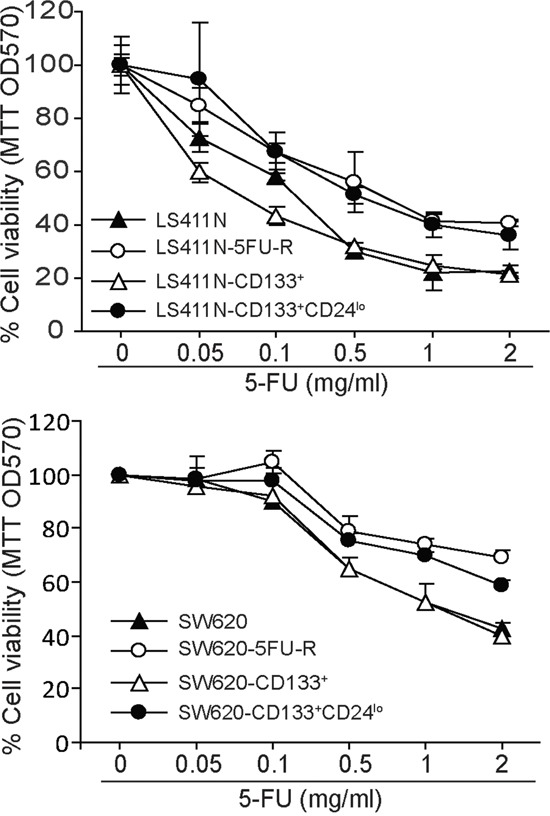
CD133^+^CD24^lo^ define a 5-FU-resistant human colon carcinoma cell phenotype The indicated four subsets of LS411N and SW620 cells were cultured in the presence of 5-FU at the indicated concentrations for 3 days and analyzed for viability by MTT assays. Cell resistance to 5-FU is expressed as % cell viability.

Both CD24^hi^ and CD24^lo^ cells have been shown to possess cancer stem cell properties [50–55]. Our above observation that CD24 expression is lower in 5-FU-resistant LS411N-5FU-R and SW620-5FU-R cells led us to hypothesize that CD133^+^CD24^lo^ cells may represent 5-FU-resistant colon CSCs. To test this hypothesis, CD24^lo^ cells were sorted from LS411N-CD133^+^ and SW620-CD133^+^ cells (Figure 5A and 5B) and examined for their sensitivity to 5-FU. Analysis of cell viability revealed that LS411N-CD133^+^CD24^lo^ and SW620-CD133^+^CD24^lo^ cells exhibit decreased sensitivity to 5-FU as compared to LS411N-CD133^+^ and SW620-CD133^+^ cells (Figure 6). LS411N and SW620 cells are advanced human colon carcinoma cells and the majority of these tumor cells have a mesenchymal cell phenotype. Both LS411N and SW620 cells exhibit high sphere formation capability (Supplementary Figure S1). We also compared sphere formation capability between the four subsets of SW620 cells and observed that the CD133^+^CD24^lo^ cells showed further higher sphere formation capability that the other three subsets of cells (Supplementary Figure S3). Taken together, our data determine that CD133^+^CD24^lo^ may represent 5-FU-resistant colon CSCs phenotypes.

### CD24^lo^ colon carcinoma cells exhibit less sensitivity to 5-FU and a mesenchymal cell phenotype

**Figure 7:**
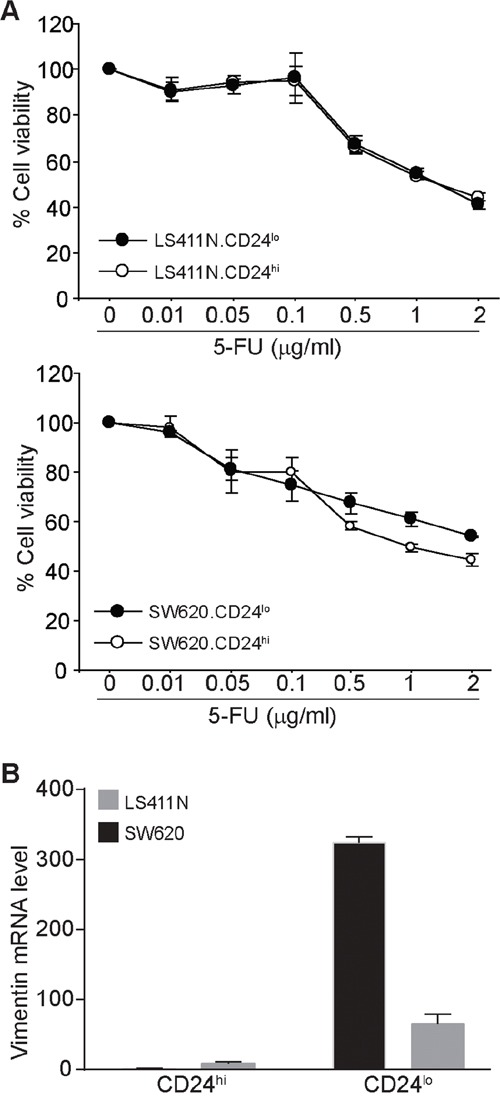
CD24^lo^ human colon carcinoma cells exhibit less sensitivity to 5-FU and a mesenchymal phenotype **A.** LS411N and SW620 cells were sorted into CD24^hi^ and CD24^lo^ cells. The sorted cells were then cultured in the presence of 5-FU at the indicated concentrations for 3 days and analyzed by MTT assay. Cell sensitivity to 5-FU is expressed as % cell viability. **B.** The sorted CD24^hi^ and CD24^lo^ cells were analyzed by real-time PCR for vimentin mRNA level. b (beta)-actin was used as normalization control.

To validate the above finding, we sorted CD24^lo^ cells directly from LS411N and SW620 cells. Analysis of sensitivity of the CD24^hi^ and CD24^lo^ cells to 5-FU indicates that the CD24^lo^ SW620 cells exhibit less sensitivity to 5-FU than the CD24^hi^ cells (Figure 7A). Furthermore, analysis of vimentin mRNA level revealed that CD24^lo^ cells in both LS411N and SW620 cells express dramatically higher levels of vimentin than the respective CD24^hi^ cells (Figure 7B).

### CD133^+^CD24^lo^ human colon carcinoma cells express higher level of ALDH

**Figure 8:**
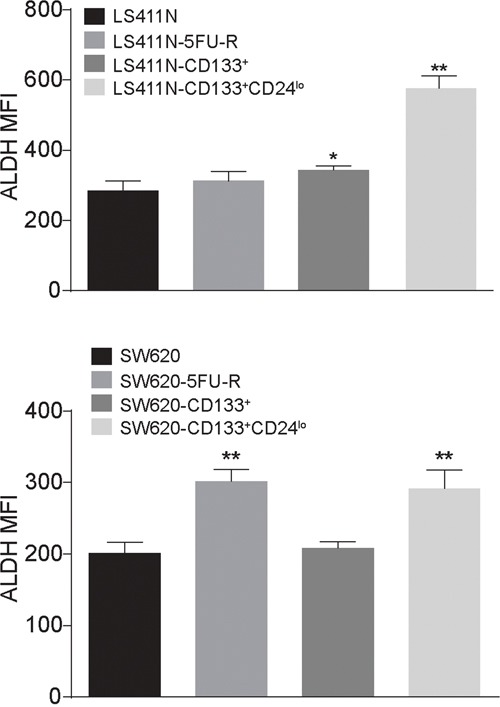
CD133^+^CD24^lo^ human colon carcinoma cells express higher level of ALDH The indicated four subsets of LS411N and SW620 cells were analyzed for ALDH enzyme level using the ALDEFLUOR Kit and analyzed by flow cytometry. ALDH protein levels were expressed as MFI. Column: mean; Bar: SD.

Aldehyde dehydrogenase (ALDH) has been proposed to be a human colon CSC marker [56, 57]. To determine whether ALDH protein level is associated with 5-FU-resistant human colon carcinoma cells, we measured ALDH protein level in the four sets of human colon carcinoma cell subsets. It is clear that ALDH is significantly higher in CD133^+^CD24^lo^ cells as compared to the parent and CD133^+^ cell lines (Figure 8).

## DISCUSSION

Human CD24 is a glycosylphosphatidylinositol-anchored membrane protein expressed on lymphocytic and myeloid cells that functions as an adhesion molecule and modulates growth and differentiation signals to these cells [58]. CD24 is also expressed on non-hematopoietic cells, including solid cancer cells [59, 60]. CD24 clearly mediates diverse functions in both hematopoietic cells and non-hematopoietic cells. In immune cells, in addition to its function in cellular growth and differentiation, ligation of CD24 also triggers a caspase-dependent apoptosis in neutrophils [61]. In non-hematopoietic cells, a large body of literatures has implicated CD24 expression in tumorigenesis and progression in a wide range of epithelial cancers including pancreatic, prostate, ovarian, breast and colorectal cancers [19, 23, 36, 50–53, 60, 62, 63]. In this study, we observed that CD24 is highly expressed on human colon carcinoma cells. However, we also observed that although CD24 is highly expressed in the human colon carcinoma cell lines, a small population (0.12-1.66%) of human colon carcinoma cells express low intensity of CD24, suggesting the heterogeneity of CD24-expressing cells in human colon carcinoma cells.

In human colorectal cancer, CD24 expression is significantly correlated to late tumor stages and lymph node metastasis. In a survival study, CD24 expression correlated significantly to shortened patient survival [62]. Consistent with its function in tumor progression and metastasis, CD24^+^ human colon carcinoma cells have been shown to possess cancer stem cell characteristics [23, 33, 36] and exhibit enhanced chemotherapy-resistance, self-renewal and tumorigenic capacity both *in vitro* and *in vivo* compared to CD24^−^ subpopulations [54]. In this study, we compared the genome-wide gene expression profiles between LS411N-CD133^+^ and LS411N-5FU-R and between SW620-CD133^+^ and SW620-5FU-R cells. Surprisingly, CD24 expression is significantly lower in the 5-FU-resistant LS411N-5FU-R and SW620-5FU-R cells than in LS411N-CD133^+^ and SW620-CD133^+^ cells. Further analysis of cell surface CD24 protein levels validated our gene-wide gene expression analysis and revealed that 5-FU treatment enriched not only CD133^+^ but also CD24^lo^ subsets of human colon carcinoma cells. However, our observation does not necessarily contradict the previous observation that CD24^+^ tumor cells are subsets of colorectal CSCs since the 5-FU-resistant human colon carcinoma cells are all CD24^+^. Instead, we further defined a subpopulation of the CD24^+^ cells as the potential colon CSCs: CD133^+^CD24^lo^ colon cancer cells.

In contrast to human colon carcinoma cells, CD24^−^ cells are proposed as breast CSCs [35, 50–53]. Combinations of CD44^+^CD24^−^ cells have been shown to possess breast CSC characteristics [53]. Furthermore, CD44^+^CD24^−/lo^ breast cancer cells are characteristics of breast chemoresistant CSCs [52]. Analysis of CD44^+^CD24^lo^ cells in human colon carcinoma cell lines indicated that six of eight human colon carcinoma cell lines contain a subset of CD44^+^CD24^lo^ cells (Supplementary Figure S4A & S4B), and the majority of CD44^+^CD24^lo^ cells are also CD133^+^CD24^lo^ cells (Supplementary Figure S4C). Therefore, CD133^+^CD44^+^CD24^lo^ may represent a subset of human colon CSCs that are responsible for human colon cancer 5-FU resistance. The scope of CD133^+^CD44^+^CD24^lo^ as human colon cancer stem cells and 5-FU resistance biomarker requires further studies since certain human colon carcinoma cells (i.e. LS411N) harbor 5-FU-resistant cell subsets but lack CD44^+^CD24^lo^ cells.

5-FU treatment of human colon carcinoma cells resulted in the generation of 5-FU-resistant cells that are enriched for CD133^+^ tumor cells, and to a lesser degree, CD44^+^ tumor cells [11], suggesting that enrichment of colon CSCs might be an underlying mechanism of colon cancer chemoresistance [11, 14, 49]. Approximately one in 262 CD133^+^ human colon cancer cells are estimated to be colon CSCs [8]. Based on these observations, we generated, by 5-FU selection in the culture medium, the 5-FU-resistant LS411N-5FU-R and SW620-5FU-R cells which happen to be CD133^+^ based on flow cytometry analysis. We also generated, by cell sorting, the LS411N-CD133^+^ and SW620-CD133^+^ cells which happen to be 5-FU-sensitive based on cell viability assay. Genome-wide gene expression profiling of these two models identified CD24, a known CSC marker in various types of human cancers [23, 33, 35, 36, 50–53], as a differentially expressed gene. Further functional studies validate that CD24^lo^ human colon carcinoma cells, in combination with CD133^+^, represent the putative 5-FU-resistant human colon CSCs. Treatment of human colon carcinoma cells with high dose of 5-FU did not increase CD133 expression level or decrease CD24 expression level (Supplementary Figure S5), suggesting that 5-FU does not regulate CD133 and CD24 expression. Taken together, we have identified a novel subset of human colon CSCs that may underlie human colon cancer 5-FU resistance.

In addition to CD24, several other genes, including genes such as LGR5 and SOX2 with known functions in human colon stem cell self-renewal and maintenance [7, 34, 50, 64], have also been identified. However, it seems that LGR5 protein level is very low in both LS411N and SW620 cells and is not significantly different between the 5-FU-sensitive CD133^+^ cells and the 5-FU-resistant CD133^+^CD24^lo^ cells (Supplementary Figure S6). Furthermore, ALDH levels appear to be positively correlated with CD133^+^CD24^lo^ cells. Therefore, CD133^+^CD44^+^CD24^lo^ALDH^hi^may further define a 5-FU-resistant human colon carcinoma stem cell phenotype from the CD133^+^CD24^lo^ cells, which also requires further studies.

Human colorectal cancers have recently been classified by the colorectal cancer subtyping consortium into four consensus molecular subtypes (CMS1, CMS2, CMS3 and CMS4) [65]. CMS4 tumors showed clear upregulation of genes implicated in epithelial-to-mesenchyal transition, tended to be diagnosed at more advanced stages, and result in worse overall and relapse-free survivals [66]. Chemoresistance, including resistance to 5-FU, and resultant tumor recurrence and metastasis accounts for more than 90% mortality in human colorectal cancer patients. In this study, we also observed that CD24^lo^ cells exhibit higher expression level of vimentin, a mesenchymal cell marker. Therefore, it is likely that the 5-FU-resistant CD133^+^CD24^lo^ cells may be characteristics as a CMS4 colon cancer, which requires further characterization. Nevertheless, we have determined that CD133^+^CD24^lo^ define a 5-FU-resistant human colon cancer stem cell phenotype.

## MATERIALS AND METHODS

### Human colon cancer specimens and cells

Human colon cancer cell lines RKO, HCT116, LS411N, COLO205, T84, HT29, SW480, and SW620 were obtained from American Type Culture Collection (ATCC) (Manassas, VA). ATCC has characterized these cells by morphology, immunology, DNA fingerprint, and cytogenetics. LS411N-5FU-R and SW620-5FU-R cells were selected from LS411N and SW620 cells, respectively, by culturing cells with increasing doses of 5-FU as shown in Figure 1A.

### Selection of 5-FU-resistant human colon carcinoma cell lines

Human colon carcinoma cell lines LS411N and SW620 cells were cultured in the presence of increasing 5-FU concentrations. 5-FU concentration were started at 0.05 μg/ml. Cells were harvested and cultured again every 7-14 days with increasing 5-FU concentrations 0.05, 0.1, 0.5, 1.0 and 2.0 μg/ml. Cells that survived 2.0 μg/ml 5-FU are maintained as 5-FU-resistant cell lines.

### Cell viability assays

Cells were seeded in 96-well plates at 2-10×10^3^ cells/well in 100 μl culture medium for 3-5 days. Cell viability assays were performed using the MTT cell proliferation assay kit (ATCC, Manassas, VA) according to the manufacturer's instructions.

### Flow cytometry

Cells were stained with fluorescent dye-conjugated anti-CD133 (Miltenyi Biotec), anti-CD44, anti-CD24 and anti-LGR5 (Biolegend) mAbs. Cells were then analyzed by flow cytometry.

### Cell sorting

CD133^+^ and CD133^−^ cells were sorted from human colon carcinoma LS411N and SW620 cell lines, respectively in the Flow Cytometry Core Facility at Medical University of South Carolina. Four cell lines were established: LS411N-CD133^+^, LS411N-CD133^−^, SW620-CD133^+^ and SW620-CD133^−^. LS411N-CD133^+^ and SW620-CD133^+^ cells were further sorted into LS411N-CD133^+^CD24^lo^ and SW620-CD133^+^CD24^lo^ cells from LS411N-CD133^+^ and SW620-CD133^+^ cells, respectively. CD24^hi^ and CD24^lo^ cells were sorted from LS411N and SW620 cells in the Flow Cytometry Core Facility at Medical College of Georgia.

### Analysis of colon cancer cell sphere formation

Tumor cells were cultured at 1×10^5^ cells/well in 200 μl DMEM medium plus 20 ng/ml EGF and 10 ng/ml basic FGF (Pepro Tech). Cells were cultured in ultra-low attachment surface polystyrene 96-well plates for 7-21 days.

### Immunohistochemistry

Human colon carcinoma specimens from patients without chemotherapy and patients after 5-FU therapy were provided by the Cooperative Human Tissue Network (CHTN, Southern Division, University of Alabama, Birmingham, AL). Each specimen and chemotherapy history were reviewed by a board-certified pathologist at the CHTN Division. Human Colon cancer tissue microarray slides were provided by CHTN (Mid-Atlantic Division, University of Virginia, Charlottesville, VA). The tissue microarrays were designed by the National Cancer Institute (NCI) pathologists and statisticians for high statistical power for examination of associations of markers with patient clinical outcomes. The tissues were stained with anti-human CD133 antibody (Miltenyi Biotec). Slides were counterstained with hematoxylin (Richard-Allan Scientific, Kalamazoo, MI). Immunohistochemical stainings were performed at the Georgia Pathology Services.

### DNA microarray

RNA was isolated from tumor cells. The quality of RNA was analyzed on a 2100 Bioanalyzer (Agilent Technologies, Santa Clara, CA) and assured by a RNA Integrity Number (RIN) ≥ 7. The Human Gene 2.0ST array (Affymetrix, Santa Clara, CA), which covers 30,654 coding transcripts, was used for the gene expression profiling. Total RNA samples were processed using the Ambion WT Expression Kit (Life Technologies, Carlsbad, CA) and GeneChip WT Terminal Labeling kit (Affymetrix). The synthesized sense strand cDNAs were then fragmented and biotin-labeled using GeneChip WT Terminal Labeling kit. The labeled cDNAs were hybridized onto the arrays using Affymetrix GeneChip Fluidics Station 450 systems according to the manufacturer's protocol. The expression data were imported into Partek GS version 6.6 using standard import tool with GC-RMA normalization. The differential expressions were calculated using ANOVA of Partek package. Differentially expressed genes were analyzed by clustering and tree view programs as previously described [67].

### ALDH protein measurement

ALDH protein levels were assessed using the ALDEFLUOR kit (STEMCELL Technologies, Cambridge, MA) according to the manufacturer's protocol. Cells were harvested, washed with ALDEFLUOR Assay Buffer, and resuspended in ALDEFLUOR Assay Buffer. Samples were stained with ALDEFLUOR Reagent (5 μL per ml of sample) and incubated for 60 minutes at 37°C. ALDEFLUOR DEAB Reagent-treated samples were used as negative controls. Cell samples were washed with ALDEFLUOR Assay Buffer and analyzed using flow cytometry.

### Statistical analysis

All statistical analysis was performed using SAS 9.2, and statistical significance was assessed using an α level of 0.05. Kaplan-Meier patient survival and cancer recurrence analyses were used to examine differences in time to recurrence by CD133 stain intensity. A log rank test was used to assess differences in survival between the groups. Student's *t* test was also used to compare differences between difference treatment groups. A *p* <0.05 was taken as statistically significant.

## SUPPLEMENTARY FIGURES


