# Supplementary material for: CD133+CD24lo defines a 5-Fluorouracil-resistant colon cancer stem cell-like phenotype
Source: Oncotarget. 2016 Sep 21;7(48):78698–712. doi: 10.18632/oncotarget.12168 (PMC5346671; doi:10.18632/oncotarget.12168)
Supplement: Supplementary file 1 [file oncotarget-07-78698-s001.pdf]

$CD133^{+}CD24^{lo}$ defines a 5-Fluorouracil-resistant colon cancer stem cell-like phenotype

SUPPLEMENTARY FIGURES

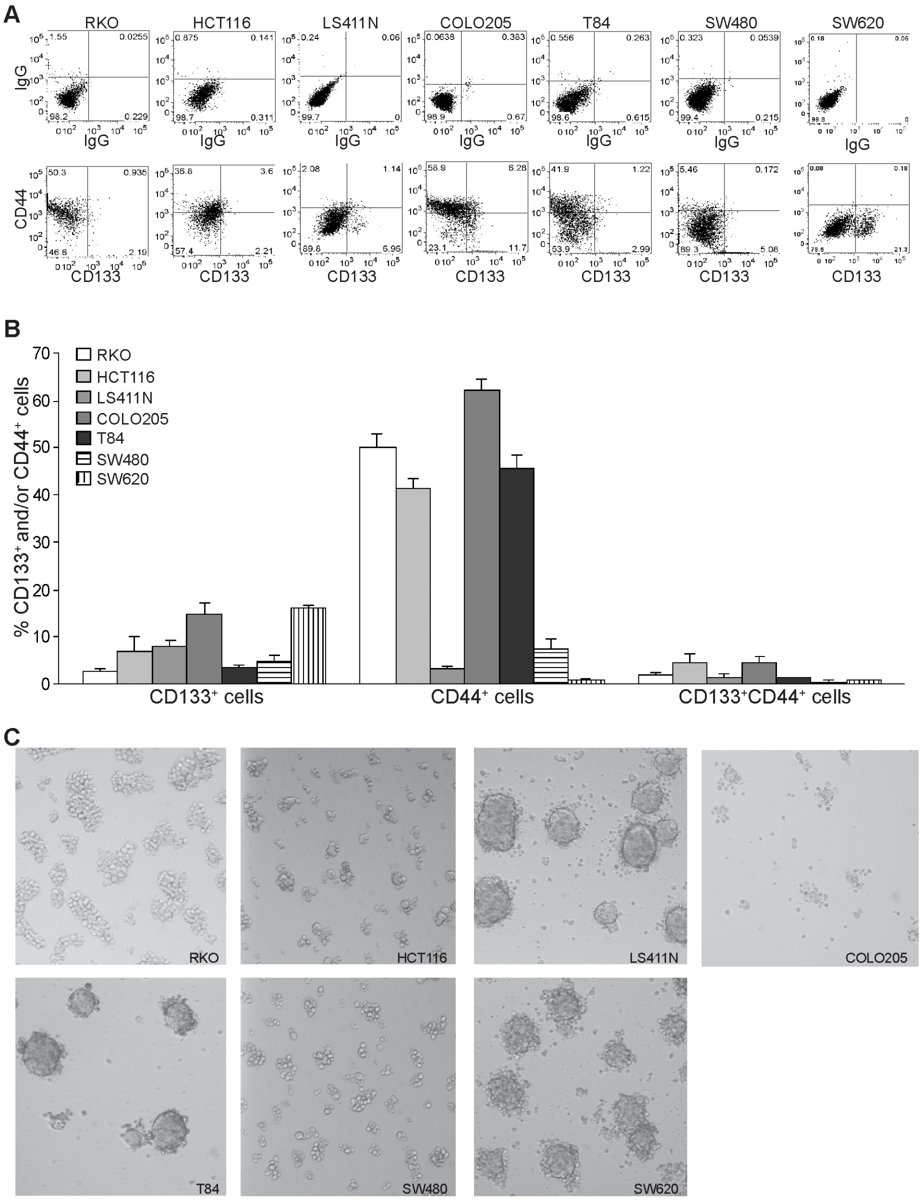

Supplementary Figure S1: Colon cancer stem cell markers and sphere formation potential of human colon carcinoma cell lines. A. Seven human colon cancer cell lines were stained with CD133- and CD44-specific antibodies and analyzed by flow cytometry. Shown are representative images of CD133- and CD44-expressing cells. **B.** The percentages of $CD133^{+}$, $CD44^{+}$ and $CD133^{+}CD44^{+}$ cells as shown in A are quantified. Column: mean; Bar: SD. **C.** Sphere formation capacity of human colon cancer cell lines. The indicated human colon cancer cell lines were cultured in the serum-free medium plus EGF and basic FGF in ultralow adhesive culture plates for 7 days. Shown are representative images of each of the cell lines.

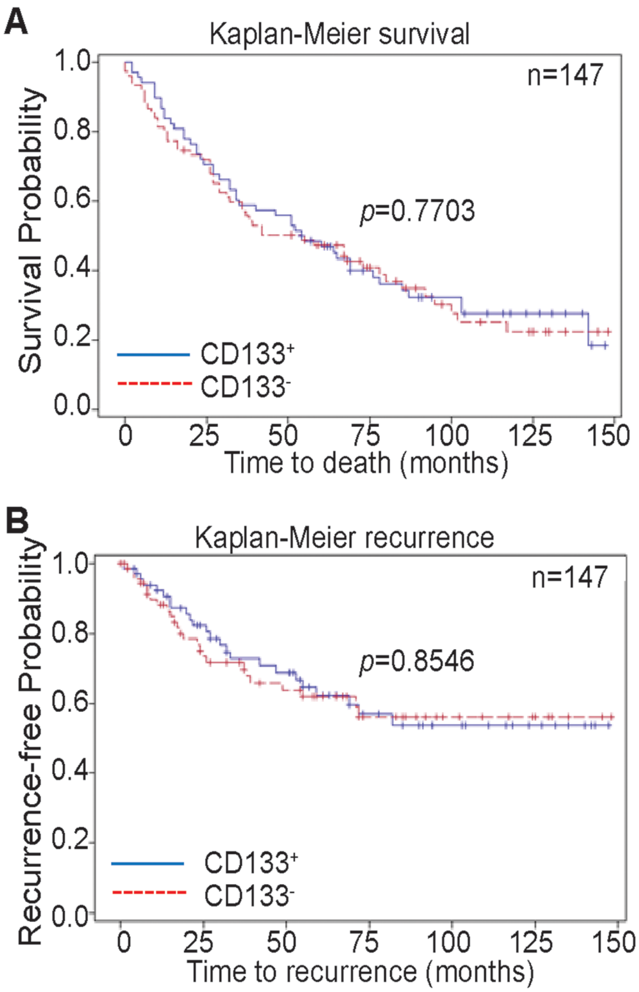

Supplementary Figure S2: Correlation between CD133 protein level and patient survival and cancer recurrence in human colon cancer patients. Tissue microarray slides containing human colon cancer specimens (n=141) were stained for CD133 protein level. The stained specimens were then graded and statistically analyzed for correlation with patient survival **A.** and cancer recurrence **B.** Each variable is indicated by colored lines in the plot.

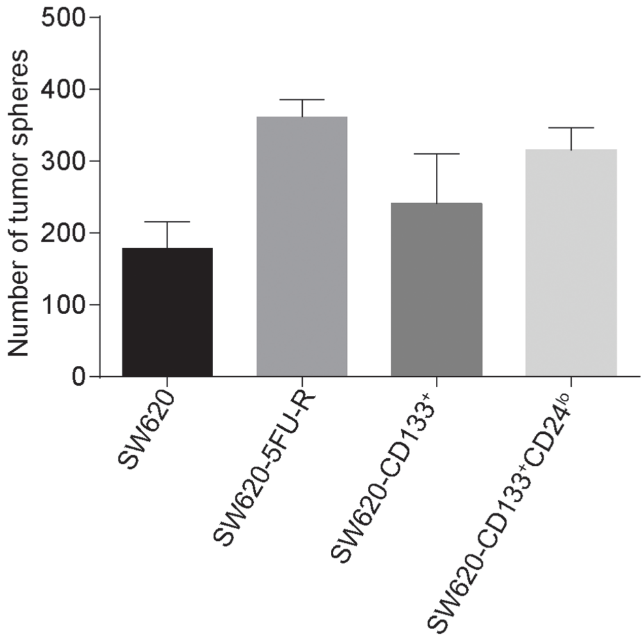

Supplementary Figure S3: Sphere formation potential of colon carcinoma cells. The four subsets of SW620 cells were cultured in ultra-low attachment 96-well plates. Spheres were counted under a microscope and expressed as number of spheres per well. Column: Mean; Bar: SD.

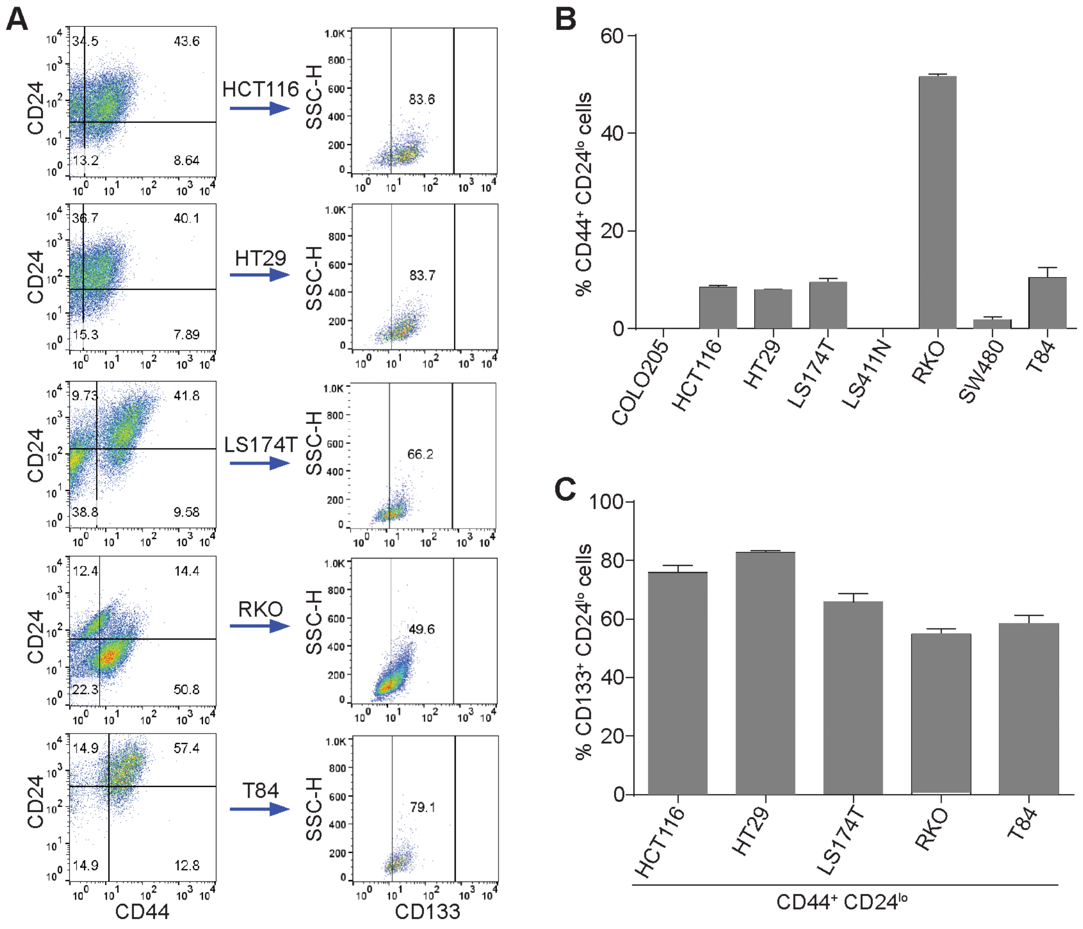

Supplementary Figure S4: CD44$^+$CD24^{lo} human colon carcinoma cells are also CD133$^+$. A. Human colon carcinoma cell cells were stained with CD44-, CD24- and CD133-specific mAbs. CD44$^+$CD24^{lo} cells were then gated and analyzed for CD133$^+$ cells. Shown are representative flow cytometry plots of the five of the eight human colon carcinoma cell lines that have CD44$^+$CD24^{lo} cells. **B.** Quantification of CD44$^+$CD24^{lo} cells in eight human colon carcinoma cells. Column: Mean; Bar: SD. **C**. Quantification of CD133$^+$ cells in the CD44$^+$CD24^{lo} cell population. CD44$^+$CD24^{lo} cells as shown in A and B were gated and analyzed for % CD133$^+$ cells in this cell population. Column: Mean, Bar: SD.

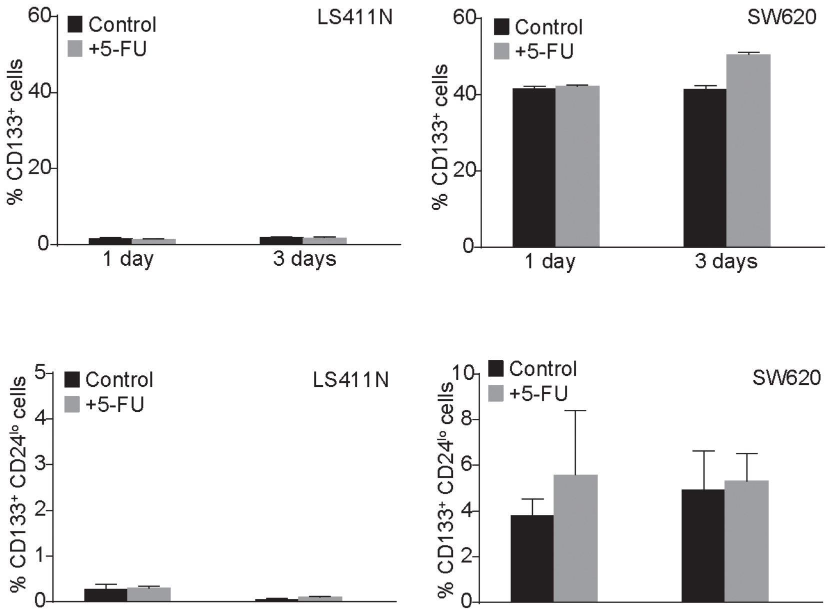

Supplementary Figure S5: 5-FU does not alter CD133 and CD24 expression level in human colon carcinoma cells. Human colon carcinoma LS411N and SW620 cells were cultured in the presence of 5-FU at the indicated concentrations. Cells were collected at the indicated time points and stained with CD133- and CD24-specific mAbs. The stained cells were then analyzed by flow cytometry. $CD133^+$ and $CD24^+$ cells were quantified. Column: Mean; Bar: SD.

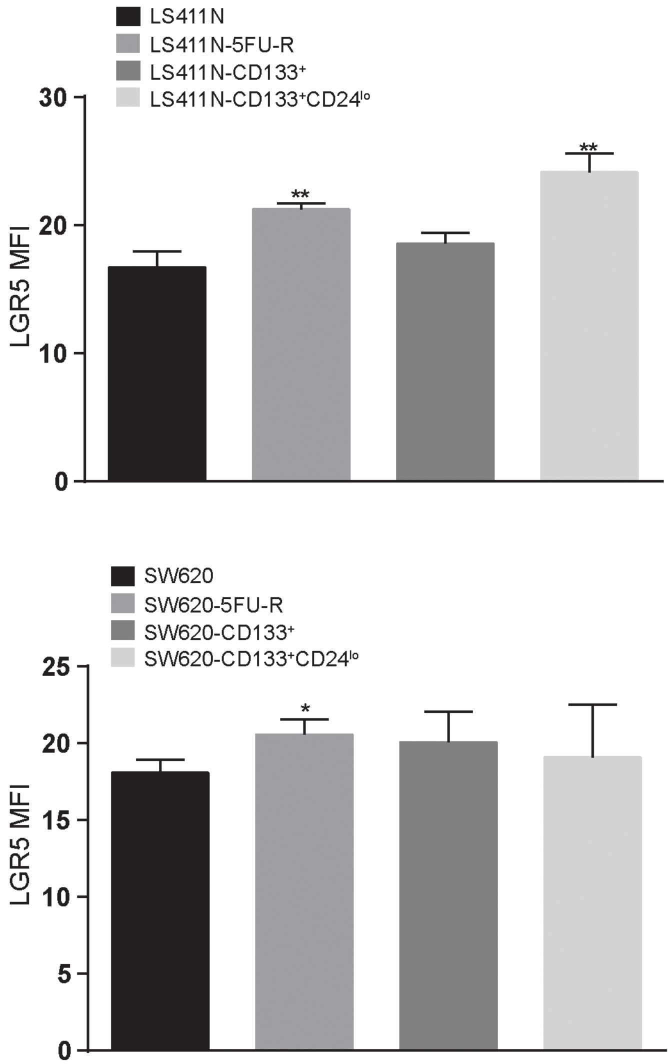

Supplementary Figure S6: LGR5 protein levels in human colon carcinoma stem-like cells. The four subsets of LS411N and SW620 cells were stained with LGR5-specific mAb and analyzed by flow cytometry. The LGR5 MFI in each cell subset was quantified. Column: Mean; Bar: SD.
